# Hydrotalcite-quinolinate composites as catalysts in a coupling reaction

**DOI:** 10.1186/s13065-016-0214-8

**Published:** 2016-10-31

**Authors:** Eloisa Ríos, Magali Hernández, Ilich A. Ibarra, Ariel Guzmán, Enrique Lima

**Affiliations:** 1 Instituto de Investigaciones En Materiales, Universidad Nacional Autónoma de México, Circuito exterior s/n, Cd. Universitaria, Del. Coyoacán, CP 04510 México, D. F. Mexico; 2 ESIQIE-IPN, Departamento de Ingeniería Química—Laboratorio de Investigación en Materiales Porosos, Catálisis Ambiental y Química Fina, UPALM Edif.7 P.B. Zacatenco, GAM, 07738 México, D.F. Mexico

**Keywords:** Adsorption, Catalysts, Hydrotalcites, Oxidation, Aluminium

## Abstract

Samples of layered double hydroxides were prepared by a sol–gel procedure. Quinolinate Al(C_9_H_6_NO)_3_ units were added during the synthesis, leading to composite quinolinate hydrotalcite-like compounds. The amount of quinolinate was varied, showing that the number of organic building blocks determines the physicochemical properties of materials, which differ significantly from those commonly reported for hydrotalcites without any quinolinate. The order of layers, specific surface area and coordination of aluminium were the parameters most significantly influenced by the presence of the quinolinate as a part of the brucite-like layers. The composite quinolinate-hydrotalcite materials were tested to catalyse the Kabachnik–Fields reaction.

**Graphical abstract:**
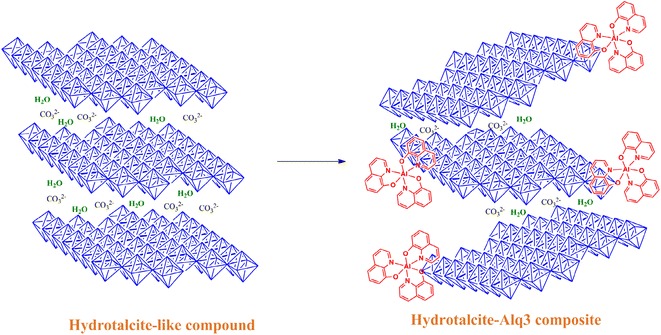
Adding of quinolinate Al(C_9_H_6_NO)_3_ to hydrotalcite-like compounds creates disorder in the stack of brucite-like layers, leading to a significant modification of structural, textural and catalytic properties. The presence of quinolinate inhibits the enchainment of octahedral blocks in hydrotalcite but develop specific surface areas as high as 600 m^2^g^−1^.

Adding of quinolinate Al(C_9_H_6_NO)_3_ to hydrotalcite-like compounds creates disorder in the stack of brucite-like layers, leading to a significant modification of structural, textural and catalytic properties. The presence of quinolinate inhibits the enchainment of octahedral blocks in hydrotalcite but develop specific surface areas as high as 600 m^2^g^−1^.

## Background

Layered double hydroxides (LDHs), also known as anionic clays or hydrotalcite-like compounds, correspond to a class of intercalation compounds [1]. The term layered double hydroxide is technically a more correct description. However, hydrotalcite-like compounds is the most frequently used term. LDHs have the ideal formula $\left[ {{\text{M}}_{{ 1 -{\text{x}}}}^{\text{II}} {\text{M}}_{{_{\text{x}} }}^{\text{III}} \left( {\text{OH}} \right)_{ 2} } \right]^{\text{x}+} \left( {{\text{A}}_{{{{\text{x}} \mathord{\left/ {\vphantom {{\text{x}} {\text{n}}}} \right. \kern-0pt} {\text{n}}}}}^{{{\text{n}}-}} } \right)\, \cdot {\text{mH}}_{ 2} {\text{O}}$. Metallic cations (M^II^ and M^III^) are located in coplanar octahedra [M(OH)_6_] sharing edges and forming M(OH)_2_ layers with the brucite structure. The partial substitution of the divalent cations by trivalent ones induces a positive charge in the layer, which is balanced by the anions between the hydroxylated layers, where water molecules are also present [2, 3]. A large number of LDHs have been synthesized by varying the nature of trivalent and divalent cations in the layers or through the intercalation of a great diversity of interlayer anions, including simple inorganic anions such as carbonate, phosphate, halides and nitrate [4] and organic anions as well as complex anions [5]. In the same manner, it is possible to synthesize LDHs containing three or more cations in the layers [6]. A unique characteristic of these layered materials is called the memory effect, which is their ability to reconstruct their layered structure when exposed to water and anion-containing solutions after losing it due to heating at a moderate temperature (400–500 °C) [7]. Nevertheless, the materials formed before and after the memory effect differ in their physicochemical properties. For this reason, the memory effect is often used to modulate the surface properties of LDHs [8]. LDHs have found applications mainly as base catalysts for many organic reactions, such as aldol condensation, Michael addition and the reduction of aromatic nitro compounds [9]. Additionally, they are helpful in providing solutions to environmental problems, for example, as reducing additives for SOx and NOx removal [10]. As multipurpose materials, LDHs have found many applications, e.g., as sorbents, anion exchangers, drug delivery carriers and PVC additives [11–13].

**Scheme 1:**
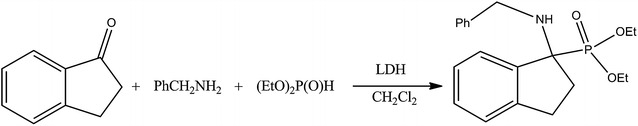
Kabachnik–Fields reaction between indan-1-one, benzylamine and diethyl phosphate to produce diethyl 1-benzylamino-2,3-dihydro-1H-inden-1-ylphosphonate

Because of the increasing number of applications where LDHs are useful, the versatility of LDHs should increase. In this sense, modification of the chemical composition of layers is a means to generate more and more efficient LDHs. Recently, the replacement of structural blocks (Al(OH)_6_)^3−^ by (AlF_6_)^3−^ was reported [14]. The addition of fluoride to hydroxide layers significantly changes the physicochemical properties of LDHs; notably, the presence of fluoride diversifies the strength and number of acid–base pairs and significantly modifies the polarity/polarisability at the LDH surface [15]. The functionalisation of LDHs, i.e., exchanging one functional group with another one, is presently accomplished by varying the nature of the metals that form a part of the layers [16]. Previously, only fluoride anions have been tested in order to replace OH structural groups, as mentioned above. Thus, the modification of LDHs by replacing OH in the layers is an unexplored but promising field. The objective of this work was to examine the effects of adding a neutral aluminium building block during the synthesis of LDHs. Some of the [Al(OH)_6_]^3−^ building blocks were replaced by quinolinate Al(C_9_H_6_NO)_3_, where the coordination number of aluminium remains 6 but the electrical charge changes. The catalytic properties of LDHs were explored in the Kabachnik–Fields reaction [17] which involves the addition of a hydrophosphoryl compound to the C=N double bond in three components: carbonyl compound, amine and phosphate. LDHs were used to catalyse the reaction to obtain diethyl 1-benzylamino-2,3-dihydro-1H-inden-1-ylphosphonate from indan-1-one, benzylamine and diethyl phosphate, as shown in Scheme 1, which deserves attention as an easy way to produce α-amino phosphonic acids [18].

## Results and discussion

### Structure and composition

Table 1 displays the formulae adjusted to the LDH composition. Note that the Mg/Al ratio is maintained close to three, as this was the nominal ratio imposed during the synthesis. However, the carbonate content is not the same for the three samples. Rather, it diminishes as the quinolinate is loaded. This result is in line with the formation of an LDH where carbonates compensate the charge of the brucite-like layers, and some of the total aluminium of the composite material exists in the block Alq3.

**Table 1:** Characteristics of samples of LDH and hybrid LDH-Alq3 prepared by sol–gel

Sample code	Mg/Al ratio	% of replaced Al(OH)_6_ units by Al(C_9_H_6_NO)_3_	Chemical formula	d_003_ (Å)
MgAl-CO_3_	3	0	[Mg_0.691_Al_0.224_(OH)_2_](CO_3_)_0.112_0.61H_2_O	8.7
q10-MgAl-CO_3_	3	10	[Mg_0.718_Al_0.233_(OH)_2_](CO_3_)_0.107_(C_9_H_6_NO)_0.021_0.78H_2_O	8.5
q30-MgAl-CO_3_	3	30	[Mg_0.721_Al_0.242_(OH)_2_](CO_3_)_0.091_(C_9_H_6_NO)_0.065_0.72H_2_O	8.4

**Fig. 1:**
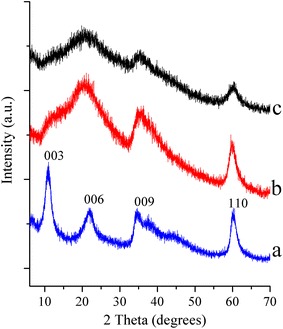
X-ray diffraction patterns of as-synthesized LDH* a* MgAl-CO_3_,* b* q10-MgAl-CO_3_ and* c* q30-MgAl-CO_3_

Figure 1 displays the XRD patterns of two LDHs containing Alq3 compared to those of Alq3-free LDH. In the absence of Alq3, the typical pattern of an LDH prepared by the sol–gel method was obtained. The peaks were broad but well-defined, confirming that the layered structure of hydrotalcite had been obtained. The following points are important to note regarding the consequences of the presence of Alq3: LDHs containing Alq3 showed a very different pattern. In the sample q10-MgAl-CO_3_, the peaks corresponding to planes (003) and (006) appeared together as a very broad peak from 10° to 30° while the reflexion (003) appeared as a weak shoulder. The peaks associated with planes (006) and (110) were resolved in all three patterns. The intercalation of Alq3 units between the interlayer space of hydrotalcite-like compounds is discarded as a possibility, as the position of the (003) diffraction peak is approximately the same in all three samples. However, it is clear that the presence of Alq3 induces disorder in the stack of brucite-like layers. This result is not surprising as the octahedral blocks Alq3 and (Al(OH)_6_)^3−^ are chemically very different in nature. The high electronic π charge and the neutrality of Alq3 inhibits the enchainment of octahedra, limiting the formation of large layers. The XRD pattern of the sample q30-MgAl-CO_3_ exclusively shows broad signals of low intensity, suggesting that the amorphous contribution is important in this sample. Indeed, the NMR results presented below suggest that a part of Alq3 accumulates, and thus, this sample tends to be a composite with a region enriched in Alq3.

**Fig. 2:**
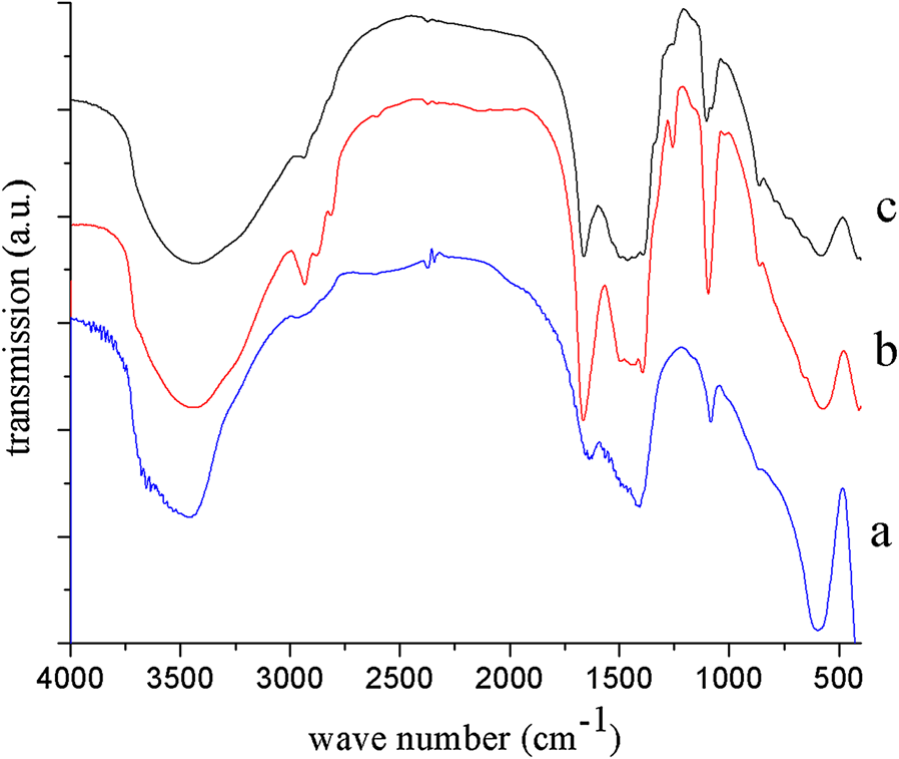
FTIR spectra of as-synthesized LDH* a* MgAl-CO_3_,* b* q10-MgAl-CO_3_ and* c* q30-MgAl-CO_3_

Following the structural characterisation, Fig. 2 shows the FTIR spectra of three LDH samples. In all three spectra, a broad absorption band is observed between 3600 and 3000 cm^−1^, which is ascribed to the $\nu_{{_{{{\text{O}}{-}{\text{H}}}} }}$ vibrational mode. Spectra of samples containing Alq3 show low-intensity absorption bands between 2750 and 3000 cm^−1^, characteristic of stretching modes of C–H bonds. Various bands corresponding to the chemical structure of Alq3 were resolved. The band close to 1450 cm^−1^ is due to $\nu_{{_{\text{CH}} }}$ of aromatic rings, while those at 600 and 950 cm^−1^ originate from the δ vibrational modes of aromatic rings [19, 20]. The C–O and C–N bonds in Alq3 are evidenced by the presence of a band at 1264 cm^−1^. It should be emphasised that the intensity of the Alq3 bands is not proportional to the amount of Alq3 in the samples, which may be explained by the different orientation of Alq3 in each sample. The NMR results below show that the samples q10-MgAl-CO_3_ and q30-MgAl-CO_3_ possess different symmetries because interactions between Alq3 and LDH are favoured when Alq3 is present in a low amount. Additionally, changes in the orientation of carbonates as a consequence of the presence of Alq3 are not clear from FTIR spectra; the $\nu_{{_{\text{CO}} }}$ of CO_3_^2−^ with a D_3h_ symmetry appears at 1412 cm^−1^ in the spectrum of MgAl-CO_3_ and at 1390 cm^−1^ in the spectra of Alq3-LDHs [21]. The shift of the CO_3_^2−^ absorption band to lower wave numbers as a consequence of the presence of Alq3 can be explained, as the π electron density inhibits the order of layers, modifing the interactions between the brucite-like layers and interlayer anions and water.

**Fig. 3:**
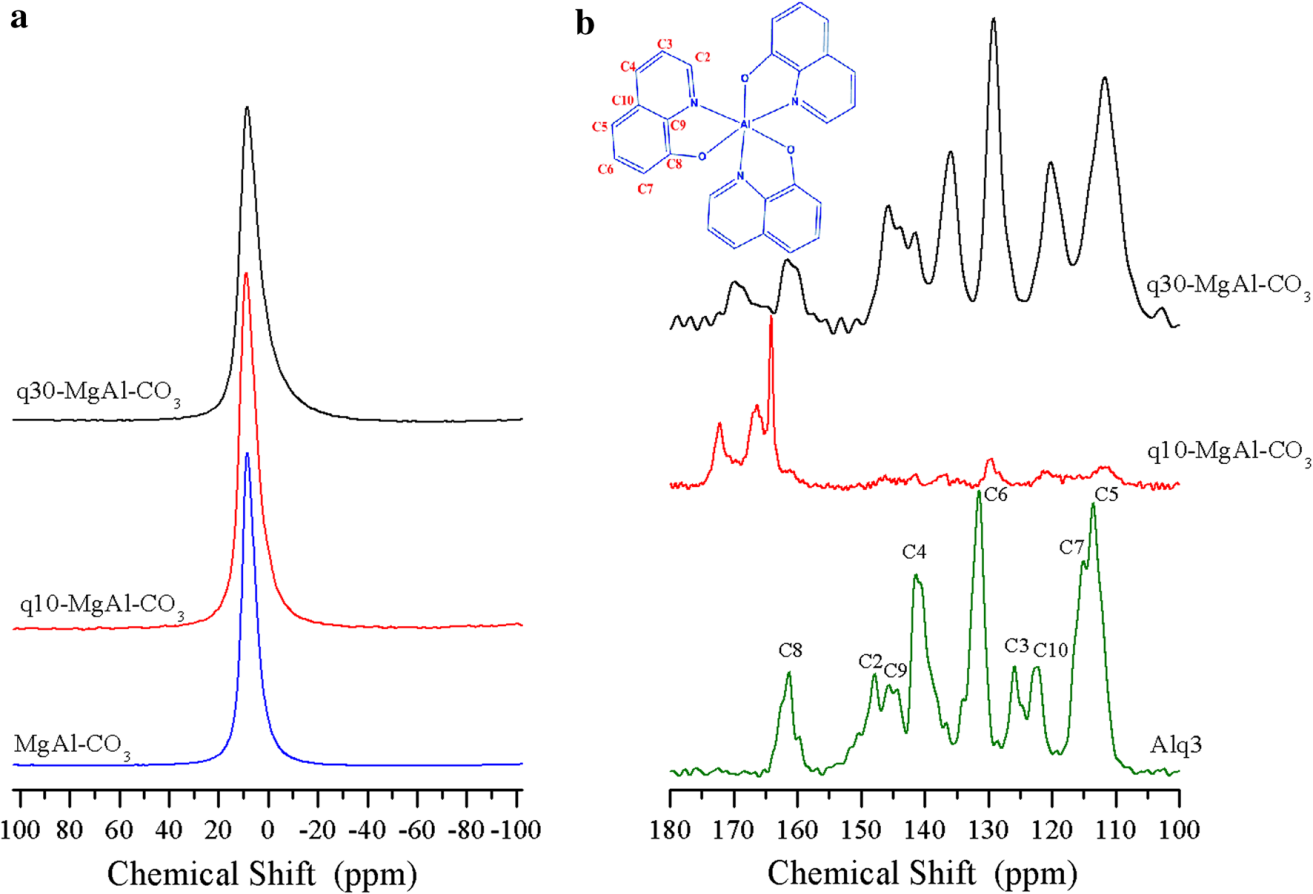
**a** ^27^Al MAS NMR spectra of as-synthesized LDH, **b** ^13^C CP MAS NMR spectra of Alq3 and Alq3-containing LDHs

The ^27^Al MAS NMR spectra included in Fig. 3a show only an isotropic peak at approximately 9 ppm, indicating that aluminium is six-coordinate. The presence of Alq3 broadens the NMR peak, in line with the heterogeneous environment of the aluminium. Figure 3b includes the ^13^C CP MAS NMR spectra as well as the reference spectrum of pure Alq3. The NMR peaks were labelled according to the carbons in the structure of Alq3, included as an inset in the same figure. The positions and intensities of the peaks match with those of isomer α Alq3, which has symmetry C_1_. When a small amount of Alq3 is incorporated into the brucite-like layers, as in sample q10-MgAl-CO_3_, relative intensities of the NMR peaks of quinolinate change. However, this is not a conclusive result about the formation of a new isomer of Alq3. The signal for carbon 8, however, appears as a double peak, suggesting that an interaction occurs between the Alq3 and brucite-like layers. The q10-MgAl-CO_3_ spectrum differs significantly from that of q30-MgAl-CO_3_ and pure Alq3. It is apparent that in the sample q10-MgAl-CO_3_, Alq3 acquires the C_3_ symmetry, which is characteristic of the γ isomer [22]. This result is relevant because the optical properties are determined by isomerism and the γ isomer is hard to find. With a high concentration of Alq3 in the LDH, as in sample q30-MgAl-CO_3_, the NMR signals of Alq3 became very similar to those found in pure Alq3, suggesting that in this sample, the Alq3 is no longer in close contact with the layers of LDH but is deposited over the surface, again favouring the isomer α [22, 23].

### Thermal properties

**Fig. 4:**
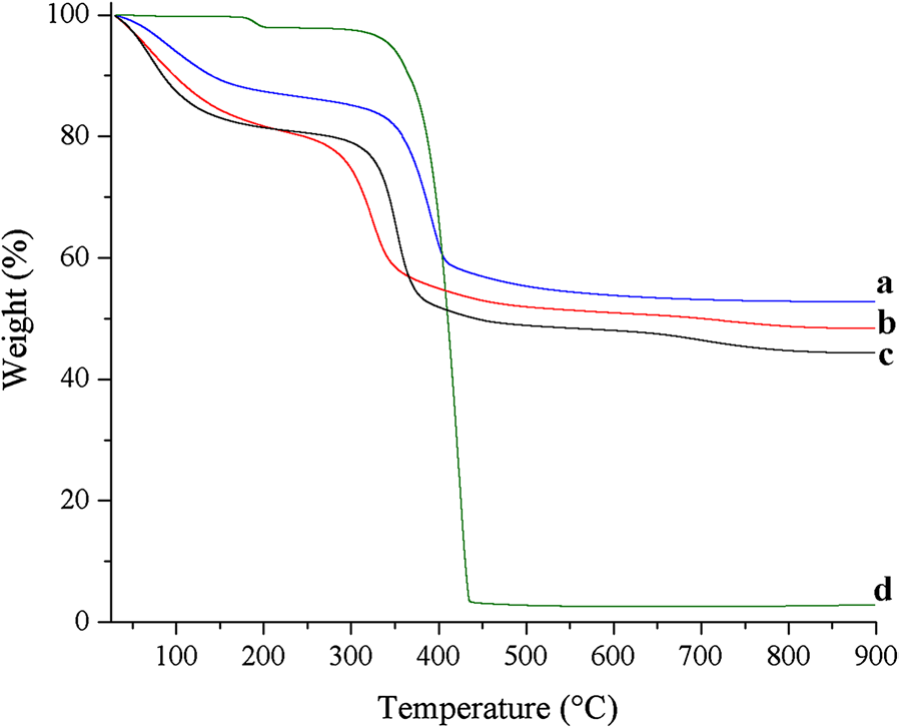
Thermogravimetric curves of the LDH samples in nitrogen.* a* MgAl-CO_3_,* b* q10-MgAl-CO_3_,* c* q30-MgAl-CO_3_ and* d* Alq3

The thermogram of Alq3-free LDH, shown in Fig. 4, is typical of a magnesium–aluminium LDH with a well-defined weight loss (12 wt%) ranging from 30 to 180 °C, corresponding to the loss of physisorbed water at the LDH surface. In this step, the weight loss percentage in Alq3 containing LDHs is 7% higher than that in Alq3-free LDH, suggesting a more developed specific surface in the presence of Alq3. This result is confirmed below in the section describing textural properties. The TG curve of Alq3 shows clearly shows that the temperature range for the decomposition of Alq3 is 350–440 °C. In this temperature range, LDH-Alq3 composites lose a higher wt% than Alq3-free LDH, consistent with the high organic content of these materials. Interestingly, the samples lose most of their organic matter in the temperature range from 327 to 436 °C; q10-MgAl-CO_3_ loses 26 wt% and q30-MgAl-CO_3_ loses 31 wt%. The samples continue to lose weight as the temperature is increased beyond 436 °C and are not thermally stable at the end of the analysis.

### Texture

**Fig. 5:**
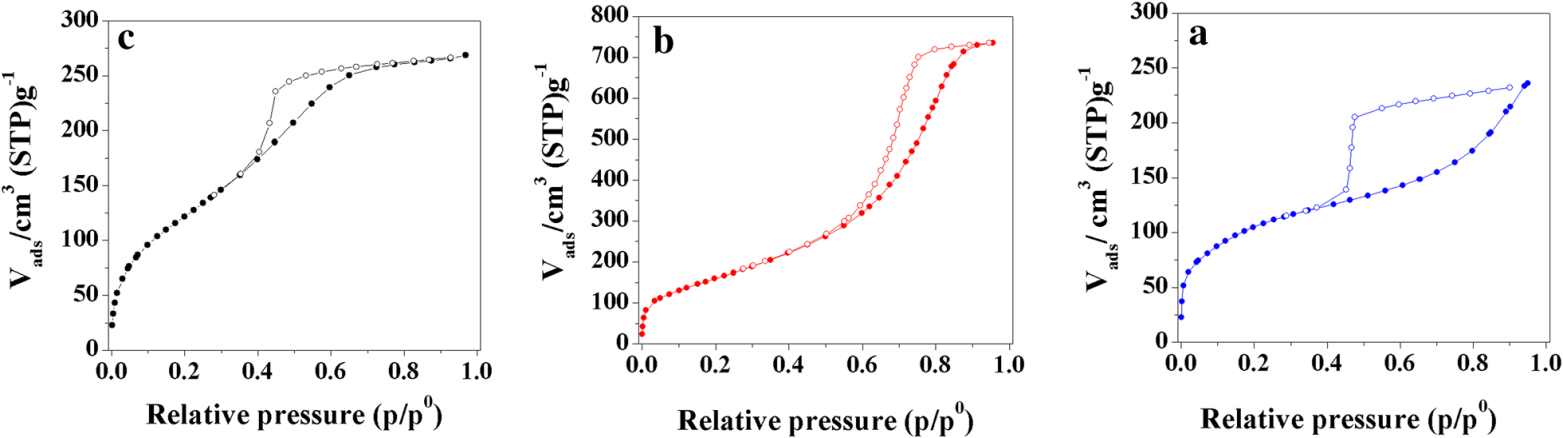
N_2_ adsorption (*filled circles*)-desorption (*empty circles*) isotherms at 77 K. **a** MgAl-CO_3_, **b** q10-MgAl-CO_3_, **c** q30-MgAl-CO_3_

The nitrogen adsorption–desorption isotherms of the samples are presented in Fig. 5. The shape of the isotherm is similar in all of the studied materials. Figure 5 displays, in all three cases, a type IV isotherm, which is characteristic of mesoporous materials [24]. In the presence of Alq3, the hysteresis loops in the relative pressure range of 0.4–0.95 fit the H2-type adsorption hysteresis, confirming the interconnectivity of pores but suggesting that the distribution of pore sizes and the pore shape is neither well-defined nor regular [25, 26], which is surely the result of the distribution of Alq3 between the LDH particles. The amount of Alq3 influences the relative pressure at which the hysteresis closes. The higher the Alq3 content is, the lower is the relative pressure, suggesting a high amount of condensation in the pores. The specific surface areas, pore volumes, and average pore sizes of the samples are summarized in Table 2. The q10-MgAl-CO_3_ sample has a considerably developed surface area (598.8 m^2^ g^−1^), but a higher amount of Alq3 has a negative textural effect. This can be related to the presence of amorphous material (evidenced by XRD results), which is probably enriched in organic compounds. Thus, the specific surface area of q30-MgAl-CO_3_ is 126 m^2^ g^−1^ lower than that of q10-MgAl-CO_3_.

**Table 2:** Textural parameters of LDH samples and hybrid LDH-Alq3 prepared by sol–gel as determined from N_2_ adsorption–desorption isotherms at 77 K

Sample code	Specific surface area (m^2^g^−1^)	Pore volume [cm^3^(STP)g^−1^]	Pore diameter (nm)
MgAl-CO_3_	310.4	71.3	2.4
q10-MgAl-CO_3_	598.8	137.6	7.1
q30-MgAl-CO_3_	472.1	108.5	3.3

**Fig. 6:**
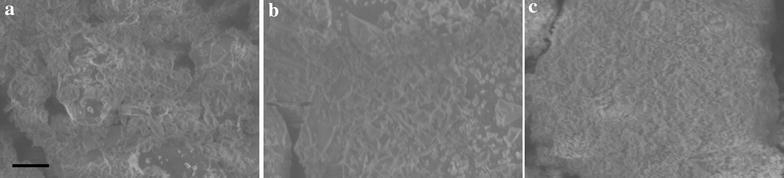
SEM images of as-synthesized LDH **a** MgAl-CO_3_, **b** q10-MgAl-CO_3_ and **c** q30-MgAl-CO_3_. *Bar* in image (**a**) is equal to 20 μm and applicable to three images

In Fig. 6, the SEM analysis demonstrates a modification in morphology of samples with the presence of Alq3. The crystals are thin and very flat, in free-Alq3 sample. Considerable variation in the crystal size is detected, ranging from about 1–8 μm. With the presence of Alq3, big particles were formed as an accumulation of smaller particles. The shape of the crystals changes, they are less flat and also the distribution of crystal size is more heterogeneous. The sample with a high load of Alq3 is seen as big aggregates of very small crystals. In this case is observed an incomplete formation of the layered structure.

### Consequences of adding Alq3 to LDH

#### Coordinative unsaturated sites of aluminium

**Fig. 7:**
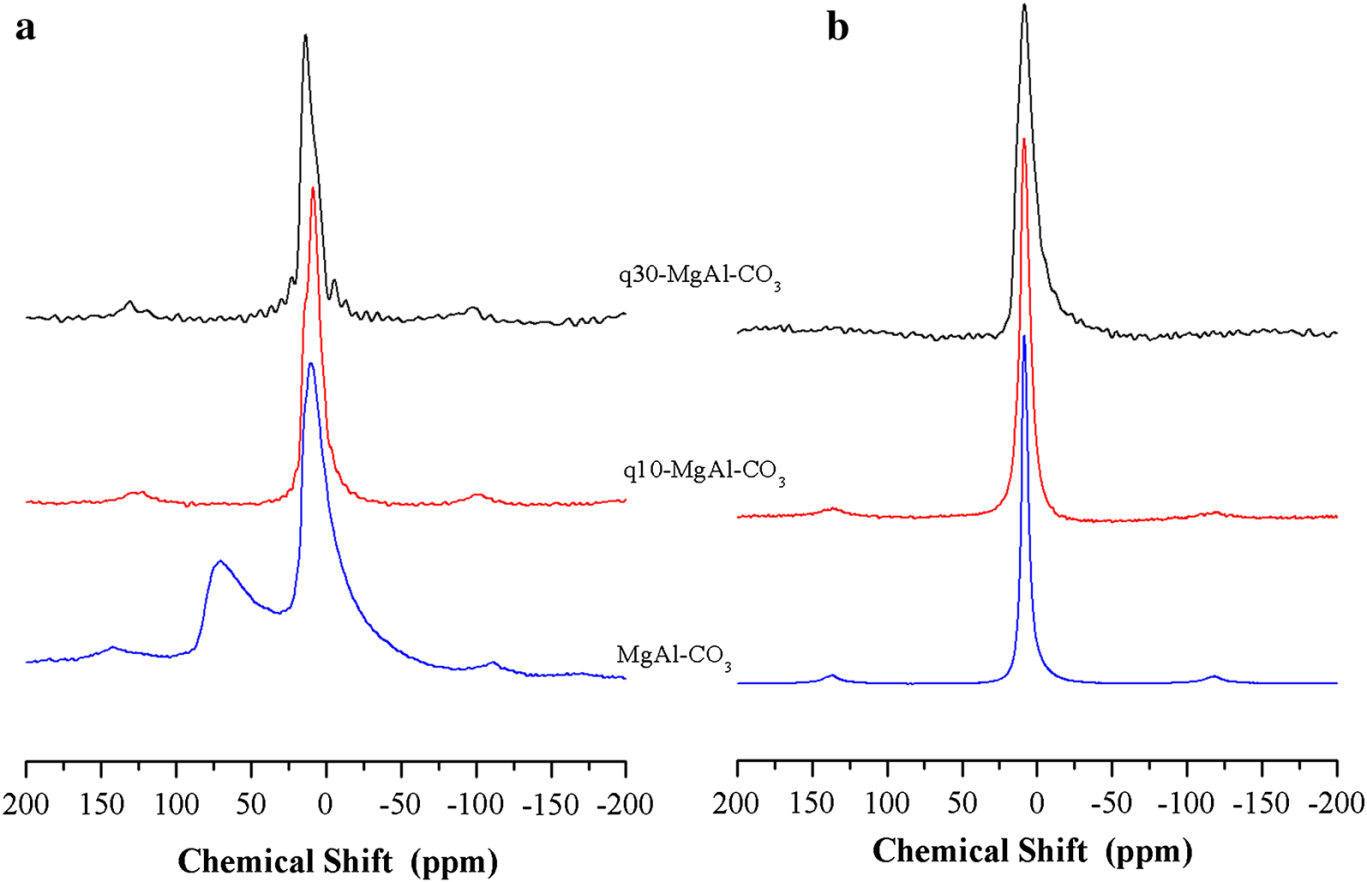
**a** ^27^Al MAS NMR spectra of LDH thermally treated at 350 °C. **b** ^27^Al MAS NMR spectra of LDH thermally treated at 350 °C and rehydrated

It is well known that when LDHs are thermally treated at moderate temperatures (350–550 °C), they lose their lamellar structure, typically leading to a mixed oxide with a periclase structure. For instance, thermal treatment of the MgAl-CO_3_ sample at 350 °C produces a mixed oxide Mg(Al)O with a periclase-like structure, confirmed by XRD (pattern not shown). As shown by the ^27^Al MAS NMR results (Fig. 7a), the collapse of the layer structure causes a change in the coordination of the aluminium atoms from 100% six-coordinate Al with oxygen ligands in an octahedral environment to Al atoms with a lower coordination, i.e., four-coordinate in a tetrahedral environment (NMR signal at 70.1 ppm), in addition to six-coordinate Al atoms in a clearly different octahedral environment, as shown by the asymmetric NMR peak close to 0 ppm [27]. This lowering in the coordination of aluminium with thermal treatment is well known in LDHs. The interesting result is that which is seen in the spectra of q10-MgAl-CO_3_ and q30-MgAl-CO_3_. The presence of Alq3 completely inhibits the presence of four-coordinate Al, even when samples are treated at 350 °C. This is the first observation in which dehydration of LDHs does not lead to coordinative unsaturated sites (CUS) of aluminium. This result is likely related to the stabilisation of the coordination of aluminium in the presence of quinolinate. This result is significant because, for example, the presence of CUS often catalyses secondary reactions such as dehydration in a more general reaction scheme for condensation [28].

The spectra of the samples that were thermally treated and subsequently rehydrated exhibit only an isotropic peak close to 0 ppm (Fig. 7b), confirming that only octahedral aluminium is present in the rehydrated samples. The peaks of the samples containing Alq3 are broader than those of the Alq3-free LDH, which is explained because of the difference in relaxation of NMR signals, cause by the presence of organics.

**Fig. 8:**
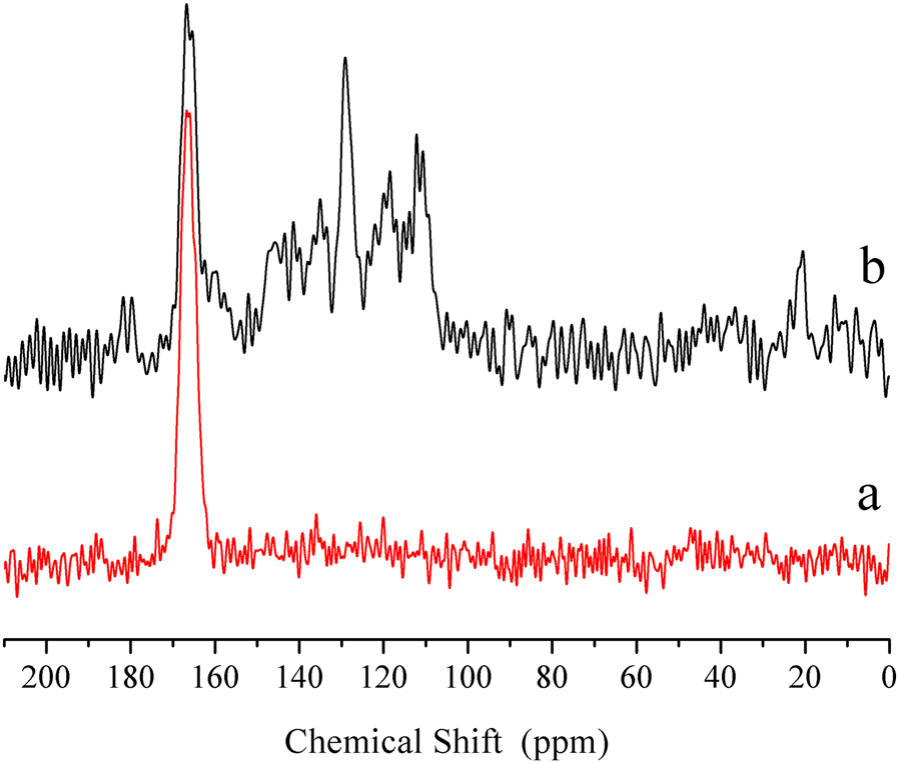
^13^C CP MAS NMR spectra of LDHs thermally treated at 350 °C.* a* q10-MgAl-CO_3_ and* b* q30-MgAl-CO_3_

Figure 8 shows the ^13^C CP MAS NMR spectra of samples containing quinolone. While the spectrum of the q10-MgAl-CO_3_ sample is only composed of a signal at 166 ppm due to carbonate species, the NMR signals due to Alq3 are well resolved in the spectrum of q30-MgAl-CO_3_ (peaks between 110 and 150 ppm), confirming that quinoline is not decomposed by the thermal treatment at 350 °C. Thus, Alq3 is a part of the LDH that could also be attached to other surfaces [29]. In q10-MgAl-CO_3_, the absence of resonance peaks associated with aromatic carbons is simply due to the low amount of Alq3 in this sample.

#### Catalysis

**Fig. 9:**
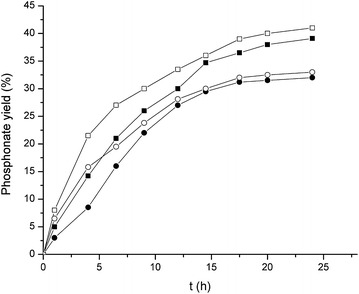
Amino phosphonate yields as a function of time from the reaction between indan-1-one, benzylamine and diethyl phosphate, using q10-MgAl-CO_3_ (*filled square* and *open square*) and q30-MgAl-CO_3_ (*filled circle* and *open circle*) as catalysts, at 30 °C (*filled square* and *filled circle*) and 45 °C (*open square* and *open circle*)

The data in Table 3 show that Alq3-free LDH and Alq3 practically do not catalyse the Kabachnik–Fields reaction. Nevertheless, as seen in Table 3 and Fig. 9, both LDH-Alq3 composites, q10-MgAl-CO_3_ and q30-MgAl-CO_3_, are able to catalyse the reaction. Reaction profiles (Fig. 9) were collected for both catalysts at 30 and 45 °C. The catalyst is active in both cases and the gain in amino phosphonate yield is not significant with the increase of temperature. The maximal yield is reached approximately 20 h after the start of the reaction. The presence of Alq3 in LDH is clearly a determining factor for the catalytic activity. The amount of quinolinate is not proportional to activity, which seems to be a result of both parameters, the part of Alq3 interacting with LDH and the specific surface developed in this LDH-Alq3 composite. Thus, q10-MgAl-CO_3_ is slightly more active than q30-MgAl-CO_3_.

**Table 3:** Yields of amino phosphonate as a function of the LDH catalyst

Entry	Catalyst^a^	Yields (%) of amino phosphonate
1	MgAl-CO_3_	2
2	Alq3	4
3	q10-MgAl-CO_3_	38
4	q30-MgAl-CO_3_	31

^a^Amount of catalyst 1 g

The amount of catalyst, of course, influences the amino phosphonate yield (Table 4). The higher the amount of catalyst is, the higher is the amino phosphonate yield under same conditions of reactants. Using 0.475 mmol of Alq3 (1.8 g of catalyst), the yield of amino phosphonate reaches 85%.

**Table 4:** Yields of amino phosphonate as a function of the amount of q10-MgAl-CO3 catalyst

Entry	Amount (g) of catalyst q10-MgAl-CO_3_	Amount (mmol) of Alq3 contained in catalyst	Yields (%) of amino phosphonate
1	0.5	0.132	12
2	1.0	0.264	38
3	1.2	0.316	55
4	1.5	0.396	66
5	1.6	0.422	73
6	1.7	0.449	80
7	1.8	0.475	85

Note that entry 4 in Table 3 corresponds to an amount of 0.77 mmol of Alq3 (in q30-MgAl-CO_3_), which is higher than entry seven in Table 4 (0.475 mmol of Alq3 in q10-MgAl-CO_3_), indicating that the catalyst is active when Alq3 is interacting with LDH, as suggested by the NMR results.

Summarising the catalysis results, the activities of the new LDH-Alq3 catalysts are close to that of aluminium phthalocyanine in catalysing this reaction under homogeneous catalysis conditions [30]. The materials available for heterogeneous catalysis of this reaction are scarce [30] and functionalised LDHs are promising catalysts.

## Experimental

### Materials

Carbonate-containing Mg–Al LDHs with a Mg/Al atomic ratio close to 3 were prepared by a sol–gel method. Briefly, aluminium tri-sec-butoxide (ATB) was dissolved in ethanol, refluxed and stirred for 1 h at 70 °C. Afterward the, temperature was reduced to 0 °C, 3 M nitric acid was added dropwise, and the mixture was stirred 1 h. Following this, magnesium methoxide (Aldrich, 99%) dissolved in butanol, and water were slowly dropped into the solution until a gel was formed, which was dried at 70 °C. In the original variant, a part of ATB was replaced by tris-(8-hydroxyquinoline) aluminium (hereafter Alq3) dissolved in dimethylformamide. The ratio of Mg/Al was maintained at 3 in the samples reported in this work. The ATB/THQA ratio was varied according to Table 1. The samples reported were not the only ones prepared, but they are representative of the changes observed in the presence of Alq3. The chemical composition reported in Table 1 is the result obtained from thermal analysis and chemical analysis conducted by inductively coupled plasma-mass spectrometry (ICP-MS), where a Thermo Scientific™ ELEMENT 2™ system was used.

### Characterisation

The samples were structurally characterised by X-ray diffraction (XRD), infrared spectroscopy (FTIR- ATR) and solid-state nuclear magnetic resonance (MAS NMR) of ^27^Al and ^13^C nuclei. The thermal and textural properties were characterised by thermogravimetric analysis (TGA) and N_2_ adsorption, respectively.

The XRD patterns were acquired using a D8 Advance-Bruker diffractometer equipped with a copper anode X-ray tube. The presence of the hydrotalcite phase and periclase structures was confirmed by fitting the diffraction patterns with the corresponding Joint Committee Powder Diffraction Standards (JCPDS cards).

A Perkin-Elmer series model 6X spectrophotometer was operated in ATR-FTIR mode in order to obtain FTIR spectra with a resolution of 2 cm^−1^.

The solid-state ^1^H-^13^C CP and ^27^Al MAS NMR single excitation spectra were acquired on a Bruker Avance 300 spectrometer. The single pulse ^27^Al NMR spectra were acquired at 78.1 MHz using a Bruker MAS probe with a cylindrical 4 mm o.d. zirconia rotor at a MAS rate of 10 kHz. The 90° solid pulse width was 2 μs, and the chemical shifts were referenced to those of an aqueous 1 M AlCl_3_ solution. The ^13^C CP/MAS NMR spectra were acquired at room temperature using a Bruker Avance 400 spectrometer operating at the Larmor frequency of 100.5 MHz, with a contact time of 5 ms, a spinning rate of 5 kHz, and π/2 pulses of 5 μs. Chemical shifts were referenced to those of the CH_2_ groups of solid adamantane at 38.2 ppm relative to TMS.

The nitrogen adsorption–desorption isotherms were determined with Bel-Japan Minisorp II equipment, using a multipoint technique. The samples were previously treated at 320 °C under vacuum for 6 h. Surface areas were calculated applying the BET equation, and pore diameter values were calculated through the BJH method.

Thermograms were recorded using a Q500HR equipment from TA instruments. Thermogravimetric curves were acquired from room temperature to 900 °C under nitrogen flux (40 ml min^−1^).

### Catalytic tests

Kabachnik–Fields reaction tests were conducted as follows: benzylamine (0.60 mmol), diethyl phosphite (0.70 mmol) and the catalyst (variable amounts were considered) were added to a solution of 1-indanone (0.58 mmol) in dichloromethane (20 mmol). The reaction mixture was stirred in a sealed vessel at 30 °C (or 45 °C) for 24 h. The catalyst was the filtered off and washed with CHCl_3_–MeOH. The course of the reaction was monitored by thin layer chromatography. The solvent was removed in vacuo, and the residue was dissolved in CHCl_3_–MeOH and purified by column chromatography on silica gel.

## Conclusion

The addition of quinolinates to brucite-like layers in LDH magnesium–aluminium-carbonates was performed through sol–gel synthesis. The presence of quinolinate Alq3 (Al(C_9_H_6_NO)_3_) is useful to develop the specific surface area and modulate the unsaturated coordinative sites of aluminium (CUS). In particular, the amount of CUS diminishes dramatically in the Alq3-containing LDHs. The LDHs with a block replacement close to 10% leads to materials with unusual specific surface area as high as 599 m^2^g^−1^, but a higher amount of Al(C_9_H_6_NO)_3_ results in a decrease of the specific surface. The LDHs-Al(C_9_H_6_NO)_3_ composites were active as catalysts in the Kabachnik–Fields reaction between indan-1-one, benzylamine and diethyl phosphate to produce diethyl 1-benzylamino-2,3-dihydro-1H-inden-1-ylphosphonate. The most active catalyst, which exhibited the highest surface area, was the LDH containing Alq3. This catalyst achieves activities similar to that of phthalocyanine under homogeneous catalysis.
